# Evolutionary Balancing of Genetic Consequence and Innovation in Mammals Through Variable Number Tandem Repeats

**DOI:** 10.1093/gbe/evaf250

**Published:** 2025-12-24

**Authors:** Petar Pajic, Omer Gokcumen

**Affiliations:** Department of Chemistry, Yale University, New Haven, CT 06511, USA; Department of Biological Sciences, University at Buffalo, The State University of New York, Buffalo, NY 14260, USA

**Keywords:** tradeoffs, mucins, function, selection, disease susceptibility

## Abstract

Understanding genomic function has historically relied on sequence conservation across evolutionary time. However, advances in genomics have revealed that functional innovations often arise from rapidly evolving, nonconserved elements that are frequently overlooked by conservation-based approaches. Among these, variable number tandem repeats (VNTRs) act as engines of both functional innovation and phenotypic consequence. VNTRs are repetitive genomic sequences whose copy numbers can vary significantly between individuals and species, influencing gene regulation, protein structure, and eventually, phenotypic diversity. Recent long-read assemblies and pangenomes now resolve VNTR loci accurately, enabling robust evolutionary reconstruction and functional associations. Here, we synthesize emerging insights into the functional and evolutionary impact of VNTRs in mammals. Specifically, we outline pressing questions on the mutational mechanisms driving VNTR evolution in humans, the selective forces maintaining their structural heterogeneity, and propose a theoretical framework for their persistence through evolutionary tradeoffs.

Significance Statement Variable number tandem repeats are highly mutable regions of the genome that have remained largely hidden due to their repetitive structure and the limitations of earlier sequencing technologies. Recent advances in long-read genomics now reveal that these sequences can shape gene regulation, protein function, and even the emergence of new biological traits. This review brings together growing evidence that such repeats are key contributors to genetic novelty and play a central role in balancing consequence and innovation throughout evolution.

## Introduction

Since the sequencing of the human genome (Lander et al. 2001; Venter et al. 2001), a central focus in evolutionary genetics has been on function. Traditional definitions of functional genomic elements have centered on evolutionary conservation, under the assumption that sequences critical to fitness are preserved by purifying selection across species. This view suggests that only a small fraction (∼5% to 7%) (Ponting and Hardison 2011) of the mammalian genome is functionally important. In contrast, advances through transcriptomic and epigenomic methods have offered a broader perspective, arguing that a much larger portion of the genome (up to 80% (ENCODE Project Consortium 2012)) may be functionally relevant. Alternative frameworks have also emerged, suggesting a “twilight zone” of functional evolution, where genomic regions may acquire functionality through variation, gene turnover, or lineage-specific innovation (Ponting 2017).

Adding to this debate, recent long-read sequencing technologies have uncovered previously inaccessible regions of the genome. These advances, led by consortia such as the Vertebrate Genomes Project (Rhie et al. 2021), the Human Genome Structural Variation Consortium (Ebert et al. 2021), and the Human Pangenome Reference Consortium (Liao et al. 2023), have enabled near telomere-to-telomere (T2T) resolution of genomes across and within species (O’Donnell et al. 2023; Wu et al. 2024; Yoo et al. 2025; Zhang et al. 2025). T2T human assemblies have revealed nearly 200 megabases (Mb) of novel sequence, particularly within pericentromeric and subtelomeric regions (Nurk et al. 2022; Vollger et al. 2022; Logsdon et al. 2025). Many of these newly haplotype-resolved regions are shaped by structural variants (SVs) and are enriched for highly repetitive elements, including centromeres (Logsdon et al. 2025), sex chromosomes (Hallast et al. 2023; Rhie et al. 2023; Makova et al. 2024), and complex loci associated with disease (Olson et al. 2023; Yilmaz et al. 2023).

In this emerging era of T2T assemblies, SVs have gained increasing recognition for their significant contributions to evolution, species diversity, and genome function (Chaisson et al. 2015; Huddleston et al. 2017 ; Pollard et al. 2018). Traditionally, SVs have included chromosomal rearrangements such as insertions, inversions, and translocations, as well as copy number variations like duplications and deletions (Conrad et al. 2010; Mills et al. 2011; Ho et al. 2020; Vollger et al. 2022; Aqil et al. 2023). Fine-scale exploration into SVs has led to several works that show their dynamic evolution and functional implications (Gökçümen and Lee 2009 ; Pajic et al. 2016 , 2019; Karageorgiou et al. 2024; Yılmaz et al. 2024; Pajic et al. 2025; Scheer et al. 2025).

**Fig. 1.**
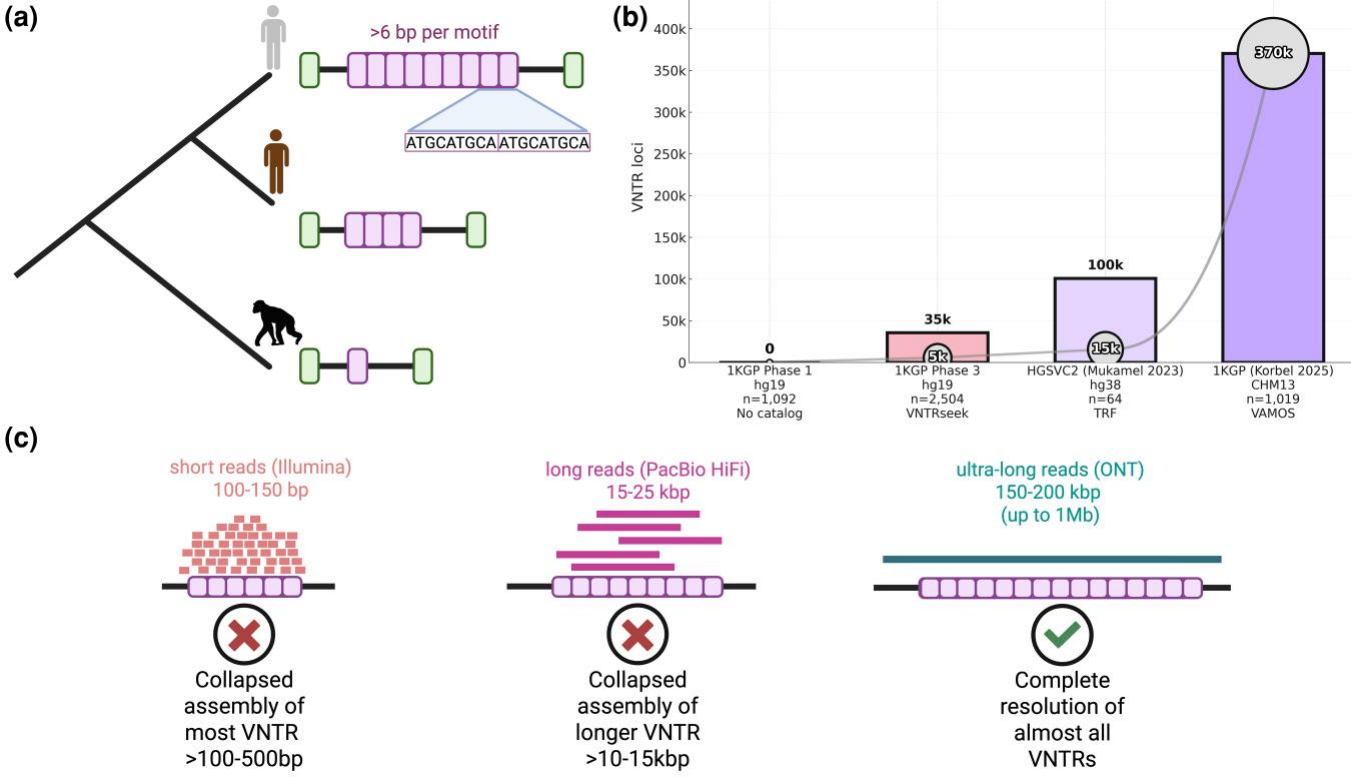
VNTR loci across major human genome sequencing efforts. a) Cladogram depicts two human individuals from different populations and a chimpanzee (silhouettes). Gene models to the right illustrate the emergence of a human-specific VNTR (magenta rectangles) within an exon. The inset shows two representative repeat units (>6 bp each), highlighting the motif structure that defines VNTRs. b) Bar plots show the total number of VNTR loci identified across major sequencing or analysis projects, with bars indicating total loci detected and bubbles denoting the subset of loci found to be commonly polymorphic. X-axis labels list the sequencing project, reference genome, human sample size, and method used. The left-most gray bar represents the negligible VNTR catalog from the 1000 Genomes Project (1KGP) Phase 1 Pilot (*n* = 1,092; hg19), which did not systematically characterize VNTRs (1000 Genomes Project Consortium et al. 2012). The following pink bar corresponds to 1KGP Phase 3 (*n* = 2,504; hg19, VNTRseek), where 35,638 VNTR loci were identified, 5,676 of which were commonly polymorphic (Eslami Rasekh et al. 2021). The lavender bar shows results from the Human Genome Structural Variation Consortia 2nd release: “HGSVC2” (*n* = 64; hg38, TRF), where 100,844 total loci and 15,653 commonly polymorphic VNTRs were reported (Mukamel et al. 2023). The right-most purple bar represents 1KGP sequenced with Oxford Nanopore and genotyped with VAMOS (Ren et al. 2023; Gu and Chaisson 2024) using the CHM13 reference genome (*n* = 1,019; 1KGP), revealing 370,468 total VNTR polymorphic loci (Schloissnig et al. 2025). c) The left image depicts short-read-based Illumina sequencing (light red rectangles; 100 to 150 bp), which cannot accurately map to the VNTR (pink array), causing collapsed assemblies for nearly all VNTRs. The middle image depicts PacBio HiFi long reads (magenta rectangles; 15 to 25 kbp), which span many but not all arrays, leading to collapsed or partially resolved assemblies for larger and more complex VNTRs. In contrast, the right image shows that ultra-long Oxford Nanopore reads (green rectangle; 150 to 200 kbp, up to ∼1 Mb) span entire repeat regions including the flaking regions (black line), enabling complete resolution of almost all VNTRs.

Most recently, attention has turned to a new frontier in understanding genome variation, centered on variable number tandem repeats (VNTRs) (Chaisson et al. 2023; Lu et al. 2023; Cui et al. 2024; Readman et al. 2024; Zhang et al. 2024; Ziaei Jam et al. 2024; English et al. 2025; Schloissnig et al. 2025). VNTRs are DNA segments composed of tandemly repeated motifs (historically defined as >6 base pairs per motif [shorter motifs are termed Short Tandem Repeats (STRs)]), where the copy number of the repeating units varies between individuals or species (Fig. 1a). With the application of improved sequencing technologies to assemble VNTR loci and the inclusion of more diverse populations by major human genome consortia, the detection and the known extent of VNTR heterogeneity have increased more than tenfold in the last decade alone (Fig. 1b).

This dramatic increase in VNTR detection is primarily a technical feat. Traditional short-read sequencing has fundamental limitations: most VNTR tracts exceed typical Illumina read lengths (Fig. 1c), leading to collapsed assemblies and widespread allele dropout (Treangen and Salzberg 2012). Even comprehensive resources such as the 1KGP Phase 1 and Phase 3 releases contain only sparse and biased documentation of VNTRs (Fig. 1b). Read-depth–based strategies and dedicated algorithms like VNTRseek (Gelfand et al. 2014) could recover only a minority of loci, systematically undercalling medium- and longer-length VNTRs. Thus, short-read-based catalogs captured only a small and highly biased subset of true VNTR diversity.

Recent advances in PacBio HiFi long-read sequencing have greatly improved VNTR resolution by providing longer, high-accuracy reads (Javadzadeh et al. 2025). In addition, emerging ultra-long-read platforms can span the largest and most complex VNTR arrays in their entirety, together with their flanking sequence, enabling accurate copy number estimation, sequence-level reconstruction, and haplotype phasing (Jain et al. 2018) (Fig. 1c). These technologies have dramatically expanded the known landscape of VNTR diversity, leading to the discovery of new rare and common disease-associated VNTRs (Cui et al. 2024; Dolzhenko et al. 2024; English et al. 2025). Nevertheless, defining a truly comprehensive tandem repeat catalog remains a substantial computational challenge, and recent work aggregating multiple detection approaches shows that each method contributes unique loci to the combined callset (Weisburd et al. 2025).

VNTRs are becoming increasingly focal in evolutionary genetics, as they are the third most common type of mutation (behind single-nucleotide polymorphisms [SNPs] and InDels) when comparing two human genomes (Collins and Talkowski 2025), and are among the most mutable loci when looking across generations (Porubsky et al. 2025). Specifically, VNTRs are emerging as promising candidates for understanding missing heritability, regulatory complexity, and the genetic basis of biological variation in general (Gymrek and Goren 2021; Ichikawa et al. 2023; Mukamel et al. 2023; Lamkin and Gymrek 2024; Manigbas et al. 2024; Tanudisastro et al. 2025). In this review, we synthesize recent literature highlighting the evolutionary and functional importance of VNTRs in mammals, with a particular emphasis on their implications in humans. We explore how these repeat regions that were largely undetectable in the earlier genomics era are increasingly recognized as dynamic elements that drive disease susceptibility, phenotypic diversity, adaptation, and genomic innovation by affecting exons, regulatory regions, and even giving rise to de novo functional elements. We argue that incorporating VNTRs challenges conventional conservation-based views of function and reveals a broader, more nuanced understanding of genomic variation and opportunities to uncover missing heritability.

## Variable Number Tandem Repeats in Function

### Exonic Variable Number Tandem Repeats

Approximately one-third of mammalian proteins harbor repetitive, predominantly tandem segments derived from VNTRs within their coding exons (Schaper et al. 2014). While most of these repeats are conserved in sequence and in copy number, a considerable portion (∼3% to 5% (Tanudisastro et al. 2025)) exhibits variation in copy number between individuals or species (Nakamura et al. 1998), hereafter referred to as exonic variable number tandem repeats (exVNTRs). Given that exVNTRs directly affect protein sequence and structure, they are highly relevant to understanding biological variation, gene function, and human health. However, despite their prevalence and direct functional implications, exVNTRs have been challenging to characterize due to the limitations of short-read sequencing. In light of advances in long-read sequencing, which can, in most cases, span the entirety of repetitive loci, accurate investigations into the evolution and function of these regions are now feasible.

**Fig. 2.**
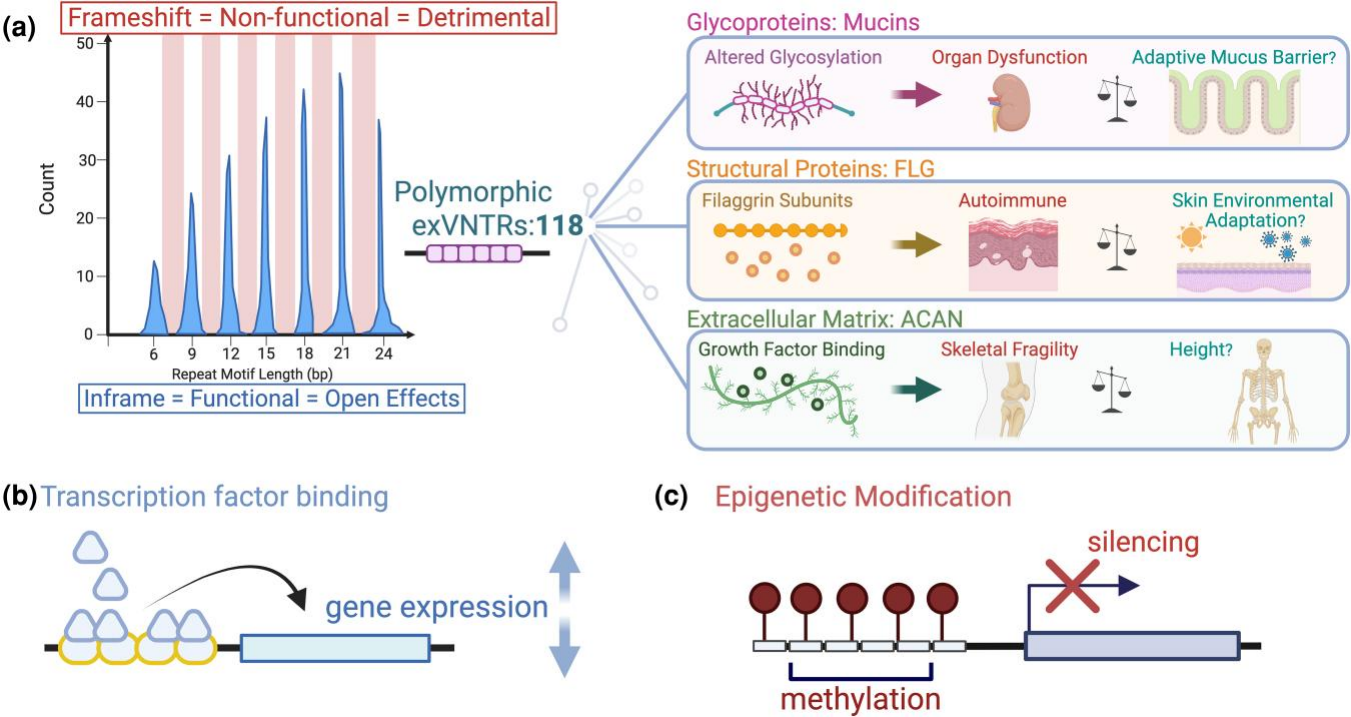
Function in exonic and noncoding VNTRs. a) Exonic VNTRs frequently occur at repeat unit lengths that preserve the reading frame (multiples of three), enabling “open” effects on protein properties, whereas frameshifted exVNTRs (red shading, non-3n motifs) typically disrupt coding potential and are removed by purifying selection. Among the 118 polymorphic exVNTRs identified with large effect sizes (Mukamel et al. 2021), many are found in glycoproteins such as mucins, where repeat copy number variation alters glycosylation and mucus barrier function in different organs, or in structural proteins like *FLG*, where variation in filaggrin subunit number can affect keratin aggregation, contributing to inflammation in atopic dermatitis or adaptation to environmental stressors. Similarly, in the extracellular matrix protein *ACAN*, exVNTR length modulates the number of chondroitin sulfate attachment sites, influencing growth-factor binding and skeletal properties. These evolutionary tradeoffs are depicted on the right separated by a scale. b) Noncoding VNTRs can influence transcriptional activity by altering the number or spacing of transcription-factor binding sites. c) ncVNTRs also affect epigenetic regulation, where changes in repeat length modify local CpG methylation and lead to allele-specific gene silencing.

exVNTRs, nested within coding exons, can directly modulate gene function by altering the encoded protein's length, domain composition, and biochemical properties (Doege et al. 1997; Gemayel et al. 2010, 2012; Marshall et al. 2021). The evolutionary relevance of these structural changes has been demonstrated through their effects on post-translational modifications, such as the availability of glycosylation sites in mucins (Higuchi et al. 2002; Reily et al. 2019; Plender et al. 2024) and on protein-protein interactions, as seen in filaggrin-keratin interface (Brown et al. 2012; Mac Donagh et al. 2024) (Fig. 2a). A critical feature observed in functional exVNTRs is that their repeat unit length retains an open reading frame (multiples of three nucleotides) resulting in tandemly repeated amino acid motifs that may be preserved under adaptive evolution (Sabino et al. 2014; Eaaswarkhanth et al. 2016; Xu et al. 2016; Pajic et al. 2022) (Fig. 2a). In contrast, frame-shifting mutations in repeats not divisible by three can truncate or dramatically alter the downstream protein sequence, potentially causing loss-of-function effects and can contribute to disease pathology (Kirby et al. 2013; Wenzel et al. 2018). Given that VNTRs can evolve rapidly, frame-shifts and their subsequent purging by selection may be relatively common. The full extent to which such specific mutations in genes with exVNTRs contribute to human disease or novel functions remains an open question, though insights from locus-specific studies are emerging.

#### exVNTRs Among Species

Several examples illustrate the functional influence of exVNTRs that contribute to trait variation across mammals. In domestic dogs, variation in polyglutamine/polyalanine encoding tandem repeats of the *RUNX2* gene, a key regulator of osteogenesis, is strongly associated with craniofacial diversity and limb proportions. Specifically, the length of the glutamine-alanine VNTR in the transactivation domain of RUNX2 correlates with short-faced (brachycephalic) versus long-faced (dolichocephalic) breeds, acting as a molecular “tuning knob” for skeletal development (Fondon and Garner 2004). In mice, *PRDM9* contains a rapidly evolving zinc finger exVNTR encoded within a single exon. The number and sequence of zinc finger repeats determine DNA-binding specificity, directing the location of meiotic recombination hotspots (Baudat et al. 2010; Altemose et al. 2017). In hybrids between *Mus musculus* subspecies, incompatibilities in PRDM9 binding caused by divergent repeat profiles contribute to meiotic arrest and hybrid sterility, a striking example of an exVNTR influencing reproductive isolation and speciation (Oliver et al. 2009; Parvanov et al. 2010).

In primates, the DUF1220 VNTR domain in the *NBPF* gene varies among nonhuman primates and is expanded in humans, increasing repeat copy number and encoded protein length. These expansions are hypothesized to contribute to increased brain size and neurodevelopmental complexity (Dumas et al. 2012). Long-read sequencing comparisons across primates have revealed over 1,500 human-specific tandem repeat expansions, 1% to 2% of which are exonic (Sulovari et al. 2019). As such, exVNTRs stand out for their capacity to drive phenotypic effects that can underlie species-specific traits (Course et al. 2021; Mukamel et al. 2021; Chaisson et al. 2023).

#### exVNTRs Within Species

More recently, exVNTRs have been shown to vary substantially when comparing human individuals and are implicated in a wide range of complex trait associations (Linthorst et al. 2020; Bakhtiari et al. 2021; Mukamel et al. 2021; Lamkin and Gymrek 2024). Mukamel et al. systematically identified 118 protein-coding VNTRs with exceptionally large effect sizes by imputing copy numbers across more than 400,000 UK Biobank individuals (Mukamel et al. 2021; Di Maio et al. 2024). These exVNTRs were found to strongly influence phenotypes such as height, hair texture, kidney function, and blood lipid levels, often with greater effect sizes than SNPs. For example, the *LPA* gene contains an exVNTR known as Kringle IV type 2 (KIV-2), consisting of tandemly repeated domains encoding kringle motifs in the apolipoprotein(a) protein. Variation in repeat number influences both isoform size and expression, where a lower number of KIV-2 repeats is associated with higher plasma lipoprotein(a) concentrations and increased cardiovascular risk due to more efficient secretion of smaller isoforms (Mukamel et al. 2021; Di Maio et al. 2024).

Many exVNTRs are associated with disease, yet these same loci often exhibit signatures of adaptive or balancing selection, suggesting that repeat variation can simultaneously contribute to pathogenic risk and confer context-dependent evolutionary advantages. In the *FLG* gene, the exVNTR encodes tandem filaggrin subunits that aggregate keratin intermediate filaments to form the cornified envelope of the skin barrier (Dale et al. 1978; Simon et al. 1996). Variation in repeat copy number directly modulates filaggrin dosage and, consequently, epidermal integrity. Shorter or truncated alleles compromise barrier function, increasing susceptibility to atopic dermatitis and ichthyosis vulgaris (Brown et al. 2012) (Fig. 2a). Conversely, maintaining variation in exVNTR length may reflect adaptive tradeoffs, where reduced filaggrin expression could enhance evaporative cooling or alter microbiome composition under certain environmental conditions (Angelova-Fischer et al. 2011) (Fig. 2a). *FLG* also shows evidence of a hitchhiking selective sweep at the nearby HRNR locus, suggesting that complex coding repeats can evolve under indirect selection while remaining tightly linked to trait-associated variation (Eaaswarkhanth et al. 2016).

In the *ACAN* gene, a 57-bp exVNTR within the first chondroitin sulfate (CS1) domain strongly influences height, with longer alleles producing taller stature by increasing the number of glycosaminoglycan attachment sites that enhance cartilage hydration and growth-factor binding within the extracellular matrix. The exVNTR alone explains up to 0.6% of height variance in individuals of African ancestry and 0.19% in Europeans, with effect sizes exceeding those of nearby SNPs (Mukamel et al. 2021). Beyond stature, shorter *ACAN* VNTR alleles are linked to intervertebral disc degeneration and osteoarthritis, likely due to reduced proteoglycan content and weaker retention of growth-factor gradients (Xu et al. 2012; Cong et al. 2018; Haddadi et al. 2022). Together, these findings suggest a continuum in which VNTR copy number tunes matrix hydration, growth-factor sequestration, and skeletal resilience (Fig. 2a).

Another striking example is the *MUC1* gene, which encodes a heavily glycosylated transmembrane mucin expressed in several epithelial tissues (Dhanisha et al. 2018). Its exVNTR spans exon 2 and consists of 60-bp tandem repeats encoding a 20-amino acid motif rich in serine and threonine, sites for O-glycosylation. Mukamel et al. found that variation in *MUC1* VNTR copy number was associated with urea levels, a marker of kidney function. Higher repeat numbers may increase glycosylation density—altering protein stability, barrier properties, or renal clearance capacity (Fig. 2a). This region is also implicated in autosomal dominant tubulointerstitial kidney disease (ADTKD) through pathogenic frameshifting insertions within the exVNTR that truncate the protein (Kirby et al. 2013; Devuyst et al. 2019). Thus, natural variation in *MUC1* exVNTR length contributes to both normal physiological variation, potentially adaptive barrier function, and disease susceptibility through effects on protein structure and epithelial integrity.

Similarly, the mucin gene, *MUC7*, which harbors an exVNTR rich in serine and threonine residues, shows evidence of local adaptation and retention of glycosylation capacity across primates (Xu et al. 2016). In another mucin, *MUC5AC*, balancing selection has been implicated in maintaining repeat copy number diversity, potentially through heterozygote advantage (Plender et al. 2024). Overall, exVNTRs have emerged as notable contributors to missing heritability and explainable variance in complex traits (Course et al. 2021; Gymrek and Goren 2021) resulting in both detrimental and beneficial effects.

### Noncoding VNTRs and Their Effect on Gene Regulation

Though historically overlooked due to their low sequence conservation and position outside of coding regions, noncoding VNTRs (ncVNTRs) are now increasingly recognized as dynamic regulators of gene expression (Zhang et al. 2024) and genome structure (Gent et al. 2011), contributing to complex phenotypes (Bakhtiari et al. 2021; Marshall et al. 2021; Mukamel et al. 2023). These effects are especially pronounced when ncVNTRs overlap with enhancers and promoters (Bellizzi et al. 2005; Vinces et al. 2009; Gemayel et al. 2012; Quilez et al. 2016; Eslami Rasekh et al. 2021), or reside within introns or untranslated regions (UTRs) (Sulovari et al. 2019; Mukamel et al. 2023). In such contexts, variation in repeat copy number can modulate transcriptional output by affecting transcription factor binding site density (Fig. 2b) (Vasiliou et al. 2012), local methylation patterns (Fig. 2c) (Quilez et al. 2016; Dolzhenko et al. 2024), and the three-dimensional architecture of chromatin (Gent et al. 2011).

#### Promoters and Enhancers

Recent studies have identified numerous ncVNTRs overlapping regulatory regions, demonstrating widespread impacts on transcriptional regulation and cellular function (Sulovari et al. 2019; Bakhtiari et al. 2021; Tanudisastro et al. 2025). A well-characterized example is the ncVNTR within the promoter of the *MAOA* (monoamine oxidase A) gene, where variation has been linked to behavioral phenotypes and risk for psychiatric conditions (Kunugi et al. 1999). Shorter alleles are associated with reduced transcriptional activity, while longer alleles drive higher expression levels of *MAOA* (Sabol et al. 1998). More recently, a second, more distal ncVNTR upstream of the canonical promoter repeat was identified and shown to regulate *MAOA* mRNA abundance, with both repeats influencing transcript variants in an isoform-specific manner (Manca et al. 2018; Marshall et al. 2021). Moreover, ncVNTR alleles at this locus show differential responsiveness to cellular stimuli, suggesting that gene-by-environment interactions may be mediated through specific repeat haplotypes. This regulatory plasticity is exemplified by studies demonstrating behavioral outcomes in response to early-life adversity: individuals carrying the low-activity (short) allele exhibit increased antisocial behavior particularly under stressful environments, whereas carriers of the high-activity allele are comparatively resilient (Caspi et al. 2002).

Few examples of ncVNTRs affecting enhancer regions have been documented, largely because enhancer landscapes remain incompletely mapped. However, it is plausible that in ncVNTRs where the repeat motif itself constitutes a transcription factor binding site, expansions overlapping with enhancer regions could have a major impact on gene expression by increasing both the number and accessibility of transcription factors bound (Vasiliou et al. 2012) (Fig. 2b). One candidate is a 72-bp VNTR located in intron 5 of the *SIRT3* gene, where both repeat copy number and internal sequence variation modulate allele-specific enhancer activity (Bellizzi et al. 2005). Longer alleles drive higher reporter gene expression, while a single nucleotide substitution within the repeat converts a GATA3 binding site into a DeltaEF1 site, abolishing enhancer function (Bellizzi et al. 2005). This highlights how both the copy number and sequence integrity of ncVNTRs can be critical for regulatory activity.

#### Introns and UTRs

Recent work has identified intronic ncVNTRs as potent regulators of gene function and disease risk (Mukamel et al. 2023). A striking example is a ncVNTR within an intron of *TMCO1*, where expanded repeat alleles are strongly associated with elevated intraocular pressure and increased risk of primary open-angle glaucoma. This repeat explains more phenotypic variance than surrounding SNPs and likely represents the causal variant underlying previously observed GWAS signals. Similarly, an intronic ncVNTR in *CUL4A* influences multiple red blood cell traits, particularly mean corpuscular hemoglobin. Beyond trait associations, this repeat also modulates splicing of *CUL4A* across multiple tissues, suggesting a direct mechanistic role in transcript regulation (Mukamel et al. 2023). Another example involves the *IL4* gene, where intron repeat variation may influence cytokine regulation (Duan et al. 2014). The shorter ncVNTR has been associated with reduced *IL4* expression and increased susceptibility to tuberculosis (Kulpraneet et al. 2019), whereas the longer allele enhances *IL4* transcription and promotes a Th2-skewed autoimmune response, advantageous in parasite-rich environments (Gyan et al. 2004; Jha et al. 2012). Together, these patterns suggest the possibility of pathogen-driven selection maintaining ncVNTR diversity to fine-tune immune responses under differing infectious pressures.

Several ncVNTRs have also been implicated in neuropsychiatric conditions such as schizophrenia and bipolar disorder (Marshall et al. 2021; Birnbaum 2023; Barlattani et al. 2024). One well-studied example is the dopamine transporter gene *SLC6A3* (DAT1), which harbors a 40-bp ncVNTR in its 3′ untranslated region (MacKenzie and Quinn 1999). The polymorphism commonly exists in 9- or 10-repeat alleles and is associated with differential expression in the striatum and prefrontal cortex, regions of the brain critical for executive function and reward processing (van Dyck et al. 2005). As such, variation in ncVNTR length has been linked to behavioral traits such as impulsivity, attention-deficit/hyperactivity disorder (ADHD), and substance use disorders (Bédard et al. 2010; Ma et al. 2016). Functional studies indicate that the 10-repeat allele generally enhances DAT1 expression and transporter function, resulting in greater dopamine reuptake and reduced synaptic dopamine, though the tissue specificity of this effect varies across studies (Mill et al. 2005 ; Vasiliou et al. 2012; Reith et al. 2022). Moreover, population-genetic analyses have revealed signatures of balancing selection across the *SLC6A3* locus (Kelada et al. 2006), suggesting long-term maintenance of both 9- and 10-repeat alleles, which may reflect adaptive tuning of dopaminergic signaling and behavioral flexibility across variable social and environmental contexts. Collectively, such findings support the hypothesis that VNTR-mediated regulatory variation contributes to cognitive traits and the evolutionary trajectory of the human brain (Sulovari et al. 2019; Course et al. 2021).

#### Epigenetic Modifications

In addition to transcriptional regulation, ncVNTRs are increasingly recognized as important mediators of epigenetics, as seen in methylation modifications. Variation in repeat copy number can significantly influence the local DNA methylation landscape, especially when ncVNTRs are positioned within CpG islands (Fig. 2c) or regions bound by histone modifiers (Dolzhenko et al. 2024). Recent high-resolution methylation profiling has begun to reveal that repeat length-dependent methylation patterns are common across the genome, particularly at disease-associated ncVNTRs (Dolzhenko et al. 2024). For example, promoter-proximal ncVNTRs can directly influence local CpG methylation and gene activity, as shown for the *EIF3H* ncVNTR, where length variation is linked to differential methylation and altered transcription (Quilez et al. 2016; Mukamel et al. 2023). These methylation changes are not transient but can be stably maintained across developmental stages and across cell types, contributing to long-term regulation of gene expression (Dolzhenko et al. 2024).

Taken together, these findings establish ncVNTRs as versatile and mostly underappreciated elements of gene regulation. By modulating transcription factor binding, splicing, and epigenetic marks, ncVNTRs influence gene expression through multiple, often interrelated, mechanisms. While most have been characterized through their deleterious associations, some evidence points to ncVNTRs as drivers of adaptive regulatory plasticity. As regulatory genomics, like ATAC-seq (Grandi et al. 2022) and FIBER-seq (Stergachis et al. 2020) continue to improve regulatory element mapping, additional examples of ncVNTRs influencing enhancer activity are likely to emerge.

### Mucins as a Model for Evolutionary Innovation

How novel gene functions evolve is a fundamental question in biology. The recent discovery of thousands of nontraditional open reading frames that encode functional proteins has significantly broadened our understanding of genomic plasticity (Deutsch et al. 2024). Researchers have begun to identify novel species-specific genes using bioinformatic methods to detect new routes to adaptive novelty (Luis Villanueva-Cañas et al. 2017) in noncoding regions across eukaryotes (Tautz and Domazet-Lošo 2011; Ruiz-Orera et al. 2014; Van Oss and Carvunis 2019; Weisman 2022; Zhao et al. 2024; Xia et al. 2025). Mobile genetic elements and SVs are well-known drivers of this process (Gemayel et al. 2010; Ji and Salzberg 2024); however, VNTRs are emerging as powerful sources of functional novelty.

Beyond impacting the function of existing genes, VNTRs can contribute to the birth of entirely new functions. A prime example is antifreeze glycoproteins (AFGPs), which evolved independently from noncoding regions in Arctic cod and Antarctic notothenioids to inhibit ice crystal formation in sub-zero waters (Chen et al. 1997b, 1997a; Baalsrud et al. 2018). In Antarctic notothenioids, AFGPs originated de novo from a trypsinogen-like pseudogene, where frameshift mutations and tandem expansions of a Thr-Ala-Ala coding motif created a novel open reading frame. In contrast, Arctic cod evolved AFGPs from a duplicated trypsinogen gene that lost its ancestral function and gained antifreeze activity through similar, albeit a different type of, repeat expansions. In both cases, antifreeze function was gained through VNTRs that serve as O-glycosylation sites, enhancing ice-binding capacity.

A more recent example of genomic repurposing is in the evolution of mucins (Pajic 2025). Mucin genes encode glycoproteins that form a critical component of mucus, providing barrier function through lubrication on organs, and defense from pathogens on epithelial surfaces (Wagner et al. 2018). They exhibit remarkable structural diversity, particularly through the presence of exVNTRs that are densely glycosylated on the encoded mucin protein (Dekker et al. 2002; Lang et al. 2007). Although some mucins have arisen through tandem gene duplications (Desseyn et al. 2000), most have arisen independently rather than through identity-by-descent, and are referred to as “orphans” (Dekker et al. 2002; Lang et al. 2007). Evidence suggests that mucin genes have undergone convergent evolution across different mammalian lineages tailored to specific physiological demands (Dekker et al. 2002; Lang et al. 2007; Pajic et al. 2022).

**Fig. 3.**
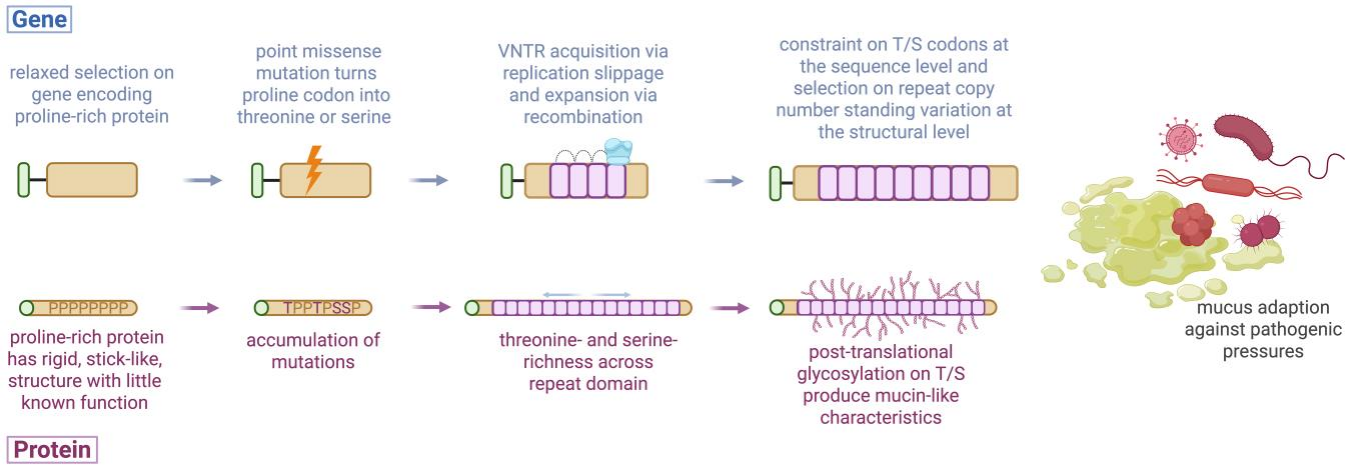
Evolutionary “mucinization” of proline-rich proteins into functional mucins. Schematic showing how proline-rich proteins can be repurposed into mucins. Top: genetic-level events. A proline-rich gene under relaxed selection (tan, with upstream exon/signal peptide in green) acquires point missense mutations (lightning bolt) converting some proline codons into threonine (T) or serine (S) codons. Replication slippage and recombination generate and expand VNTR in-frame arrays encoding T/S-rich motifs (pink). Sequence constraint maintains T/S-rich functional codons, while standing copy number variation becomes a target of selection in certain environments. Bottom: protein-level consequences. Proline-rich proteins with rigid, low-complexity domains (tan) accumulate T/S amino acids, expand into repeat-TS-rich regions, and undergo dense post-translational O-glycosylation, yielding mucin-like biophysical properties. This transition generates immediate functional heterogeneity, and once a new mucin is recruited, repeat copy number polymorphism arises as a natural consequence, opening functional flexibility that can be acted upon by natural selection, for example, through adaptive modulation of mucus properties in response to pathogenic pressures.

Pajić et al. explored the dynamic evolution of mucin genes across mammals (Pajic et al. 2022). By analyzing whole-genome assemblies from diverse species, it was demonstrated that mucin genes can evolve de novo by acquiring VNTRs encoding (P) proline-, (T) threonine-, and (S) serine-rich motifs, characteristic of mucin functional domains. Astonishingly, non-VNTR genes encoding proline-rich proteins (PRPs) recurrently evolve into mucins, establishing a new model where PRP genes serve as natural precursors, as their proline content is one mutational step from T/S peptides that enable glycosylation (Fig. 3). Furthermore, PRPs may have vestigial functions or evolve under relaxed selection, making them more permissive to evolutionary innovation. These VNTR expansions were found to be recurrent and evolving rapidly in the saliva across multiple species, likely shaped by selective pressures related to host-pathogen interactions and mucosal defense involving glycans in the oral cavity (Cross and Ruhl 2018; Wagner et al. 2018; Barnard et al. 2020; Yang et al. 2025).

The de novo birth of mucin genes and the extensive copy number variation within their VNTR domains offer valuable insights into the mechanisms of functional evolution. This variation has immediate consequences for protein size, glycosylation potential, and interactions with the extracellular environment, features that directly impact mucosal biology. As such, mucins serve as powerful models for investigating how VNTRs contribute to phenotypic diversity and adaptive evolution (Gemayel et al. 2010, 2012). Together, these findings reveal how VNTRs not only modify existing genes but also catalyze the emergence of entirely new ones, raising a central question of how such mutable yet functional elements persist through evolutionary time.

## Conclusions and Future Perspectives on Evolutionary Maintenance of VNTR Variation

As we have discussed, the apparent lack of conservation across VNTRs reflects an ongoing evolutionary interplay of both phenotypic consequence and functional innovation, often involving the same genes (Fig. 2a). Different parts of the genome experience distinct selection regimes, and VNTRs reflect this at different levels. In regulatory regions, copy number may be selected when altering the density of transcription-factor binding sites confers advantageous expression changes, whereas in coding domains, purifying selection constrains sequence integrity across motifs to preserve biochemical function (Pajic et al. 2022). Interruptions or degenerative repeats, which can modulate mutability by disrupting expansion mechanisms such as replication slippage or nonallelic homologous recombination (NAHR), may also be selectively favored (Sulovari et al. 2019). Selection can therefore act not only on repeat copy number but also on the degree of sequence homogeneity among repeat units. The interplay between these pressures shapes VNTR heterogeneity at multiple levels, framing how different evolutionary forces maintain or erode repeat variation across the genome.

While some VNTRs are under selective constraint, most evolve under **Neutrality.** Neutral regions subject to drift or relaxed purifying selection accumulate mutations without major fitness costs, leading to increased divergence among individuals and species (Hunt et al. 2011). A non-VNTR example is the reduction of olfactory receptor genes in humans relative to other primates, likely reflecting relaxed selection accompanying a decreased reliance on smell as vision became dominant (Gilad et al. 2003; Veilleux et al. 2023). Similarly, neutral alleles can persist if their deleterious effects emerge only after reproductive age, as in late-onset Huntington's disease (Ross and Tabrizi 2011; Handsaker et al. 2025). Such relaxation may also foster evolutionary innovation, as neutral or mildly deleterious mutations occasionally acquire adaptive value and become targets of positive selection (Wang et al. 2017; Zhao et al. 2023). It stands to reason that the majority of the variation seen in VNTRs may have little functional impact as they occur outside of genes, and thus evolve largely under neutrality. Yet a central question remains: why do VNTRs with clear molecular, cellular, or organismal effects, remain so variable, and which evolutionary forces maintain this diversity?

### Positive Selection

Promotes the rapid diversification of sequences that confer adaptive benefits, leading to lineage-specific functional innovations. Antimicrobial peptides such as defensins exemplify this pattern, evolving rapidly in response to species-specific pathogen pressures (Ganz 2003). Another classic example in humans is lactase persistence, where selection on SNPs near the *LCT* gene enabled continued lactase expression into adulthood, aligning genetic change with the cultural adoption of dairying in different geographies (Tishkoff et al. 2007; Kerdoncuff et al. 2025). Other work has discussed inconsistencies in the lactase story (Ségurel and Bon 2017), underscoring that detecting positive selection is difficult, even for SNPs, for which the majority of modern population genetics tools are designed.

Most common human SNPs predate the emergence of anatomically modern humans, reflecting deep coalescent times and a low mutation rate (∼10⁻⁸ per bp per generation). As a result, SNPs rarely introduce new adaptive alleles on timescales relevant to rapid Holocene environmental change (Hernandez et al. 2011; Scally and Durbin 2012). In contrast, VNTRs mutate orders of magnitude faster (∼10⁻^5^ to 10⁻^2^ per locus per generation; (Porubsky et al. 2025), continually generating novel alleles and maintaining abundant standing variation. Because VNTR alleles can behave neutrally when selection is weak, substantial repeat copy number and sequence diversity can accumulate without major fitness costs, forming a reservoir of alleles that can be co-opted when strong ecological or cultural pressures arise. These properties make VNTRs a more plausible substrate for recent, rapid human adaptation than SNPs and help explain why VNTR loci show population-specific expansions, contractions, and functional effects (Hannan 2018; Sulovari et al. 2019; Mukamel et al. 2021). Yet, despite this evolutionary potential, direct evidence for positive selection on VNTRs remains limited, partly because tools to robustly analyze selection in such loci are not available.

Detecting selection on VNTRs remains challenging, as classical scans rely on biallelic SNPs and therefore miss the multiallelic, rapidly mutating nature of VNTRs. Consequently, locus-specific analyses incorporating haplotype-level information remain the most reliable approach. A recent study of *MUC19* found striking differences in VNTR copy number between global populations and admixed Americans that carry an introgressed archaic haplotype (Villanea et al. 2025). Positive selection was then inferred using SNP-based statistics across the introgressed region, including population-branch statistics (PBS), and demonstrated via demographic simulations that these signals cannot be explained by neutrality. Earlier works on the *DRD4* VNTR used intra-allelic haplotype diversity, linkage disequilibrium (LD) decay, and allele-age estimation to argue for positive selection on the 7-repeat allele (Ding et al. 2002; Wang et al. 2004). These studies have essentially conducted traditional population genetic analyses on SNPs that are on the same haplotype harboring putatively selected VNTR alleles.

Looking forward, the field urgently needs population-genetic tools that can detect selection on VNTRs with confidence. Several gaps are holding us back. First, we need approaches that can robustly infer VNTR copy number variation in both modern and ancient genomes from short-read data. Second, we lack formal tests to evaluate whether the striking absence of frameshift mutations during exVNTR expansion deviates from neutral expectations, and whether this pattern reflects purifying selection preserving the reading frame. Third, LD calculations must be adapted to explicitly model recurrent mutations and homoplasy (van Leeuwen et al. 2015). Finally, we need mutational models that move beyond simple stepwise accumulation and instead incorporate gains and losses of multiple repeat units through recombination events.

Closing these gaps will enable integrated simulation-based and empirical frameworks to evaluate selection on VNTRs. Such approaches will jointly model copy number variation across ancient and present-day populations, reconstruct ancestral states and allele-frequency spectra, quantify temporal shifts in repeat-copy distributions, and detect selection acting both on the motifs/nucleotide content of repeat units and on haplotypes carrying specific VNTR alleles. Together, these advances will allow rigorous tests of classical positive and balancing selection, while also supporting more nuanced models, such as oscillating or frequency-dependent bouts of selection acting on standing VNTR variation in population- and time-specific ways.

### Balancing selection and evolutionary tradeoffs

Can sustain genetic variation within populations. Classic examples include the extreme polymorphism of major histocompatibility complex (MHC) genes maintained by pathogen-mediated balancing selection (Hedrick 2006). Recent work shows that even gene-disrupting SVs can persist, such as the growth hormone receptor (*GHR*) exon 3 deletion, which enhances prenatal growth in some contexts but increases risks for metabolic dysfunction in others (Allison 1954; Saitou et al. 2021; Nikkanen et al. 2022; Aqil et al. 2023). These cases illustrate how alleles can be maintained when their advantages and disadvantages are context-dependent. Within this framework, VNTRs emerge as particularly informative loci: they are highly mutable, recurrently evolving, and often pleiotropic in function. Their enduring variation likely reflects an inherent capacity to mediate both constraint and innovation, a balance that shapes genomic and phenotypic diversity. Yet, unlike well-characterized loci such as *MHC* or *GHR*, the dual adaptive and deleterious consequences of VNTR variation have rarely been examined in an integrated comprehensive manner across studies.

**Fig. 4.**
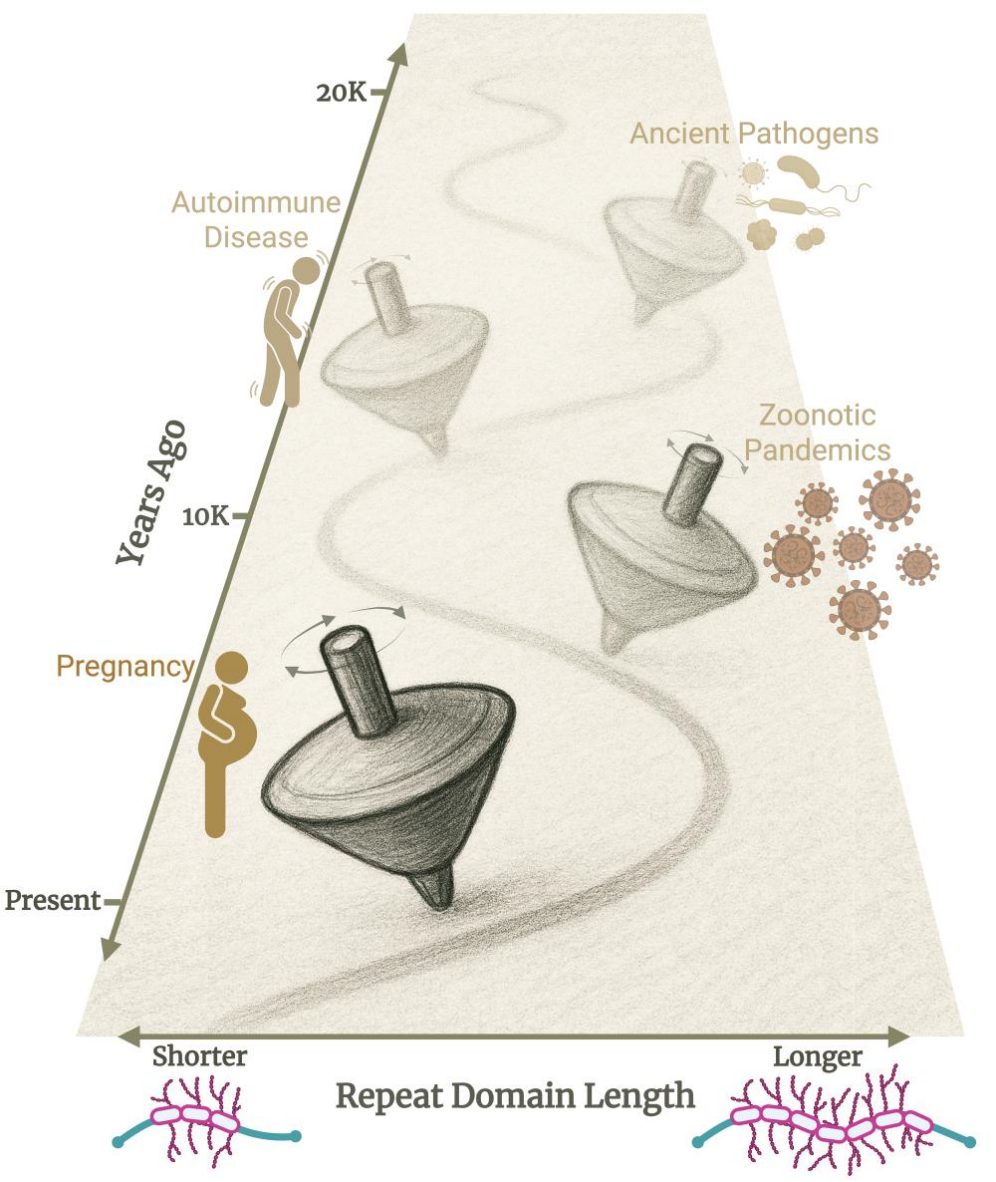
Spinning-top model of evolutionary tradeoffs. The vertical slanted-axis represents time, with the present at the bottom and the past toward the top. The horizontal-axis denotes VNTR domain length in a mucin, ranging from shorter (left) to longer (right). The spinning top illustrates the dynamic, multidimensional nature of VNTR evolution, oscillating through time and across environments as repeat copy number population allele frequencies shift configurations continuously. Bronze pictograms mark major environmental or physiological transitions (e.g. pathogen exposure, zoonotic immune challenge, pregnancy) that influence the direction and stability of the spinning top's balance. For example, pandemics may drive selection on longer mucin VNTR alleles generating stronger mucus barriers on organs against pathogens, but at the same time, increasing mucosal and immune-related disease susceptibility. Whereas shorter mucin VNTR alleles—driven by reduced autoimmune disease and improved pregnancy success through embryo implantation dependent on the mucus barrier—may be favored under benign exposure contexts.

Evolutionary tradeoffs are often depicted as a simple scale balancing benefit and cost. For pleiotropic VNTRs, however, the balance of effects can shift dramatically across tissues, environments, and historical contexts, and is further complicated by the presence of multiple alleles—each defined by different repeat copy numbers—rather than a simple two-allele system. We envision this using a “spinning-top” moving along a path through time (Fig. 4). The path represents the temporal trajectory of the allele, while the horizontal position of the top reflects which repeat configuration (shorter or longer) is favored at that moment. The tilt of the top indicates the strength of the selective bias toward that configuration, and the orientation captures which specific pressure (e.g. pathogen exposure, immune activation, pregnancy) is exerting that pull. At any given point, the same VNTR allele may confer a functional benefit in one context while simultaneously imposing a biological consequence in another, such that gain and cost can coexist rather than occur sequentially. As different ecological or physiological forces rise and fall, the top shifts position, tilt, and orientation, illustrating how VNTRs experience a continually reweighted balance of pressures rather than a single, static tradeoff. This shifting, multidimensional equilibrium helps maintain standing VNTR diversity and contributes to their rapid evolutionary trajectories. We hope that this conceptual model provides a blueprint for emerging new perspectives that incorporate the multiallelic nature of VNTRs within a pleiotropic context.

Mucin genes, discussed thoroughly in this review, offer a striking empirical framework for exploring these dynamics, as recurrent emergence and extreme variation (Prodanov et al. 2025) highlight both the instability and adaptive potential of these loci. For example, specific repeat copy numbers have recurrently evolved in the *MUC7* gene in humans and have independently expanded across mammalian lineages, likely in response to pathogenic pressures encountered in the oral cavity (Xu et al. 2016, 2020). Likewise, recent analyses of *MUC5AC* indicate that distinct common copy number alleles are maintained by balancing selection in some human populations (Plender et al. 2024).

Immune-related genes have been shaped by selection during historical pandemics, such as the Black Death (Klunk et al. 2022). It follows that earlier zoonotic pathogens, emerging alongside the agricultural revolution (Jones et al. 2013), may likewise have played major roles in shaping key immune and barrier-related genes. Accordingly, mucins, central to the mucus barrier and pathogen defense (Wagner et al. 2018), are likely to have been subjected to similar selective pressures. Alleles that conferred protection under one pathogen regime may now predispose individuals to immune hyperreactivity or autoimmunity, similar to immune-related genes such as *ERAP2* (Klunk et al. 2022). This continual evolutionary arms race of “push-pull” between pathogen-driven diversification and physiological constraint may explain the extent and potential benefit of standing VNTR variation. Consistent with this, pathogen-binding VNTRs in immune genes such as *CD209L* show population-specific signatures of balancing selection, and reveal that pathogen pressures can directly shape repeat-length diversity (Barreiro et al. 2005). Therefore, mucins, such as MUC1, could possess longer glycosylated repeat domains that strengthen mucosal barriers against pathogens but may affect important processes in different adaptive or consequential directions, such as immune (dis)regulation or embryo implantation during pregnancy (Brayman et al. 2004; Dharmaraj et al. 2009) (Fig. 4).

Future studies leveraging high-resolution genomic, transcriptomic, and proteomic tools will further clarify these dynamics. In particular, comprehensive exploration of the *mucinome* (Malaker et al. 2022; Lowery et al. 2024; Finn et al. 2025; Steigmeyer et al. 2025), linking VNTR diversity in mucin genes to corresponding glycosylation maps, will be essential for understanding how genetic structural variation modulates molecular recognition, barrier function, and microbial interactions. Integrative analyses across molecular layers promise to reveal how VNTR-driven diversity shapes adaptation, constraint, and disease susceptibility, offering a broader view of how repetitive sequences influence mammalian biology and evolution.

## Data Availability

No data was generated for this study.
